# The Impact of Imputation on Meta-Analysis of Genome-Wide Association Studies

**DOI:** 10.1371/journal.pone.0034486

**Published:** 2012-04-05

**Authors:** Jian Li, Yan-fang Guo, Yufang Pei, Hong-Wen Deng

**Affiliations:** 1 School of Public Health and Tropical Medicine, Tulane University, New Orleans, Louisiana, United States of America; 2 School of Biomedical Engineering, Southern Medical University, Guangzhou, People's Republic of China; 3 Center of System Biomedical Sciences, University of Shanghai for Science and Technology, Shanghai, People's Republic of China; 4 Laboratory of Molecular and Statistical Genetics and the Key Laboratory of Protein Chemistry and Developmental Biology of Ministry of Education, College of Life Sciences, Hunan Normal University, Changsha, Hunan, People's Republic of China; University of Texas School of Public Health, United States of America

## Abstract

Genotype imputation is often used in the meta-analysis of genome-wide association studies (GWAS), for combining data from different studies and/or genotyping platforms, in order to improve the ability for detecting disease variants with small to moderate effects. However, how genotype imputation affects the performance of the meta-analysis of GWAS is largely unknown. In this study, we investigated the effects of genotype imputation on the performance of meta-analysis through simulations based on empirical data from the Framingham Heart Study. We found that when fix-effects models were used, considerable between-study heterogeneity was detected when causal variants were typed in only some but not all individual studies, resulting in up to ∼25% reduction of detection power. For certain situations, the power of the meta-analysis can be even less than that of individual studies. Additional analyses showed that the detection power was slightly improved when between-study heterogeneity was partially controlled through the random-effects model, relative to that of the fixed-effects model. Our study may aid in the planning, data analysis, and interpretation of GWAS meta-analysis results when genotype imputation is necessary.

## Introduction

Genome-wide association studies (GWA studies or GWAS) using high-throughput genotyping data are a powerful tool and are of a great help in identifying susceptibility loci for human complex traits and common diseases [1]–[4]. However as most of these susceptibility loci have small effects, large sample sizes are usually required for having sufficient statistical detection powers. Such sample size requirement can be beyond the capacity of a single GWA study.

A partial solution to this issue is meta-analysis, which combines data from multiple studies of relatively small sample sizes, with the expectation to detect genes underlying susceptibility loci with greater power and produce more precise estimation of genetic effects, and hence to provide more convincing conclusions than the original individual studies do [5]–[8]. This strategy has been applied to and improved our understanding in a number of common diseases, such as Parkinson's disease [9], type 2 diabetes [10]–[12], bipolar disorder [13], colorectal cancer [14], and rheumatoid arthritis [15], demonstrating the applicability and usefulness of meta-analysis of GWAS.

A useful tool in GWAS is imputation, which can provide the same set of SNPs across individual studies by inferring millions of untyped/missing SNPs from typed SNPs and based on the known knowledge such as haplotype structure from HapMap [16]. Imputation can improve the power for GWAS in a single study [17], and can also be used for meta-analysis of GWAS by combining data from different studies and/or with different genotyping platforms. However, imputation is not perfect, and errors and uncertainty can be introduced in the imputed genotypes. These issues may consequently affect the detection power of meta-analysis with imputation, which however has not been fully investigated. Thus, it is necessary to investigate the impact of imputation on GWAS, for appropriate planning, data analysis, and interpretation of meta-analysis of GWAS.

To better assess the usefulness and limitation of the meta-analyses of GWAS using genotype imputation, several critical questions need to be answered:

- Does genotype imputation affect (or create) between-study heterogeneity? If yes, how does this heterogeneity caused by imputation affect the performance of meta-analysis of GWAS?
- Does imputation-based meta-analysis of GWAS with a much larger sample size always have greater power than that of individual component studies with smaller sample sizes?
- What to do in the presence of potential negative impacts of genotype imputation on the meta-analysis of GWAS?

In order to address these questions, we performed comprehensive simulation studies based on the empirical GWAS dataset from the Framingham Heart Study (FHS). We found that genotype imputation may cause between-study heterogeneity and reduce the power of meta-analysis of GWAS. Strategies were proposed to alleviate this negative impact of genotype imputation on meta-analysis of GWAS.

## Results

### Imputation effects on between-study heterogeneity

**Figure 1:**
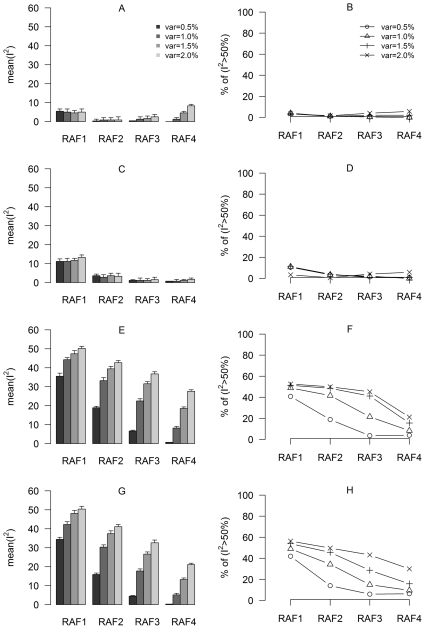
Assessment of between-study heterogeneity under various scenarios. The plots in the left column (A, C, E, G) show the mean values of 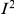, and those in the right column (B, D, F, H) show the average percentage of simulations with 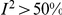 (large between-study heterogeneity). The plots in rows 1–4 are for scenarios 1–4, respectively. Descriptions of scenarios and RAF's are given in Table 2, and “var” values indicate the simulated QTL variance.

Data structure such as sample size and simulation parameters were indicated in Tables 1 and 2 (see the Materials and Methods section for details). We assessed the impact of genotype imputation on between-study heterogeneity using two measurements for the index 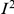: the mean value of 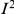 and the average percentage of simulations with 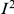
>50% (referred as large between-study heterogeneity [6]). Results were based on 1,000 simulations for each scenario with various QTL variances and risk increasing allele frequencies (RAFs) (Fig. 1). Between-study heterogeneity was almost ignorable for Scenario 1 (SNPs fully genotyped across sub-samples, Fig. 1 A and B) and Scenario 2 (SNPs fully imputed across sub-samples, Fig. 1 C and D), with the highest mean 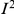 below 15% and only limited fraction (<10%) of the simulations having 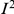
>50% for relatively rare SNPs (RAF between 0.01–0.05). In contrast, significantly higher mean 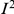 and higher percentage of large between-study heterogeneity were observed in Scenarios 3 and 4 (when SNPs were imputed in one or two sub-samples, Fig. 1 E–H). For Scenario 3, up to 60 percent of the simulations showed 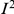
>50%, and the mean 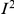 values reached 40–50% for SNPs with RAF1-3. Scenario 4 showed similar level of between-study heterogeneity to that of Scenario 3. In addition, in Scenarios 3 and 4, between-study heterogeneity was observed to increase with a larger variation explained by QTLs, and decrease with a higher RAF. Therefore, imputation may cause between-study heterogeneity, especially when imputation was performed in some but not all of the sub-samples.

**Table 1:** Selection strategy and quality control parameters for sub-population construction.

*Samples*	*Selection strategy*	*Sample size*		*No. of SNPs surviving QC*
		Pre-QC	After-QC	
Sample 1	Singletons, all unrelated subjects from the 1^st^ generation (two at most) in each pedigree, plus married-ins in the 2^nd^ and the 3^rd^ generations	2,200	2,023	412,432
Sample 2	One subject from the 2^nd^ generation in each pedigree	1,071	1,055	416,800
Sample 3	One subject from the 3^rd^ generation in each pedigree	812	806	417,532

Parameter values for quality control (QC): minor allele frequency >0.01, Hardy-Weinberg equilibrium test p-values>0.0001, sample call-rate >0.90, and SNP call-rate >0.90.

**Table 2:** Simulation schemes and parameters.

*Genotypes of the causal SNPs*	
Scenario 1	Directly-typed in all three sub-samples
Scenario 2	Imputed in all three sub-samples
Scenario 3	Imputed in Samples 1 & 2 but typed in Sample 3
Scenario 4	Imputed in Sample 1 but typed in Samples 2 & 3

*Range of risk increasing allele frequency (RAF)*	
RAF1	0.01<RAF≤0.05
RAF2	0.05<RAF≤0.10
RAF3	0.10<RAF≤0.20
RAF4	0.20<RAF≤0.50
QTL variation	0.5%,1.0%,1.5% and 2.0%

### Comparison of performance of meta-analysis with and without genotype imputation

The estimated type-I error rates and the power of meta-analysis with both fixed-effects model and random-effects model are shown in Table 3. For Scenarios 1 & 2, which have little between-study heterogeneity, both fixed-effects and random-effects models had correct type-I error rates that were below the target level 5% under all conditions. For Scenarios 3 & 4, under which considerable between-study heterogeneity existed, random-effects model still had comparable type-I error rates. The fixed-effects model however may have inflated type-I error rates, which can be over 60% greater than that of random-effects model, showing the need of taking caution in selecting appropriate meta-analysis strategy.

**Table 3:** Mean power and type-I error rate of 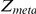 (α = 10^−7^).

	*QTL variance (%)*	*Fixed-effects model*				*Random-effects model*			
		RAF1	RAF2	RAF3	RAF4	RAF1	RAF2	RAF3	RAF4
Scenario 1	0	3.01	2.98	3.10	2.97	2.86	3.05	2.89	2.89
	0.5	18.77	20.93	19.38	19.41	16.35	20.93	19.34	19.40
	1.0	84.96	82.32	81.66	80.43	78.39	81.81	81.36	80.23
	1.5	98.79	97.76	95.69	95.90	93.24	94.66	93.89	92.29
	2.0	100.00	98.88	96.69	96.72	96.17	95.77	95.59	95.09
Scenario 2	0	3.27	3.33	3.25	3.42	3.13	3.12	3.04	3.05
	0.5	17.20	14.01	13.71	8.91	17.20	14.01	13.60	7.72
	1.0	69.90	67.77	56.32	49.29	69.90	67.77	56.00	44.66
	1.5	88.20	84.35	81.19	68.76	88.09	84.35	80.77	62.83
	2.0	91.51	90.22	86.61	73.87	91.29	90.11	85.87	66.15
Scenario 3	0	5.57	5.49	5.48	5.50	3.55	3.52	3.53	3.52
	0.5	10.13	7.42	7.65	4.04	10.92	8.14	7.65	5.70
	1.0	37.26	34.81	29.76	26.48	38.18	37.70	36.69	33.27
	1.5	46.98	44.8	41.34	34.92	46.08	47.14	46.09	42.62
	2.0	49.13	47.27	44.00	38.12	53.33	52.54	51.26	49.17
Scenario 4	0	5.56	5.53	5.52	5.56	3.54	3.53	3.56	3.54
	0.5	8.60	7.55	7.21	4.40	8.72	7.83	7.61	4.99
	1.0	32.77	35.02	35.02	31.12	33.25	32.41	32.12	30.22
	1.5	45.27	45.73	45.73	39.61	46.76	46.34	45.01	40.63
	2.0	47.50	47.89	47.89	40.94	51.43	50.20	49.74	45.55

Descriptions for Scenarios 1–4 and RAF ranges are given in Table 2. Mean power and type-I error rates were estimated based on 1,000 simulations.

The power of the meta-analysis was different for various scenarios (Table 3). In general, the highest power was observed when causal SNPs were genotyped in all individual component studies (Scenario 1), followed by the situation when causal SNPs were imputed in all individual component studies (Scenario 2), and the lowest power was seen when SNPs were genotyped in some sub-samples and imputed in the other sub-samples (Scenarios 3 & 4). For the independent populations generated through HAPGEN2 [18], Scenario 2 had similar powers as those of Scenarios 3 and 4 (results not shown). When compared between meta-analysis with fixed-effects model and that with random-effects model, the power was almost the same for Scenario 1 or Scenario 2. For Scenarios 3 & 4, meta-analysis using random-effects model performed slightly better than that using fixed-effects model, particularly when QTL variance was relatively large. For instance, when 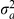 = 2%, a 5–25% power increase was shown for random-effects model over that for fixed-effects model. These results indicate that meta-analysis with random-effects model is more appropriate for GWAS with imputation.

**Figure 2:**
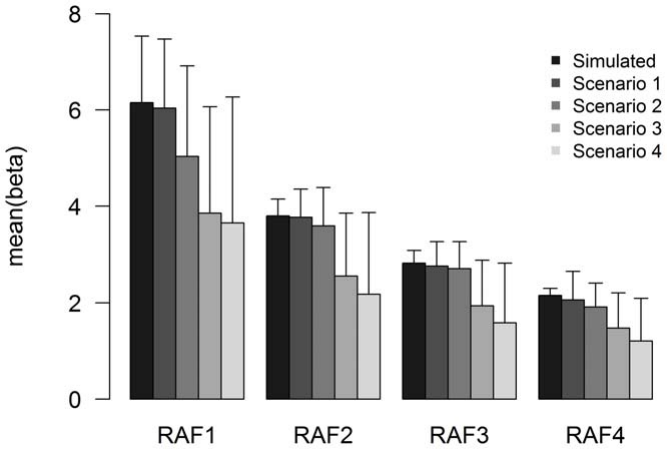
Comparison of effect size and standard error estimated by meta-analysis to simulated true values. The simulated QTLs explain 2.0% of the total trait variance.

The estimation accuracy of the genetic effects, measured by 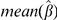 and 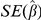, were different for various scenarios, as shown in Fig. 2. The 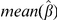 values were almost equal to the true values for Scenario 1, and were underestimated for Scenarios 2–4. The percentages of underestimation were similar across different RAF intervals. For example, when the simulated 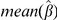 was 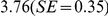 within RAF2, the 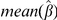 was 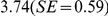 for Scenario 1, 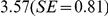 for Scenario 2 and 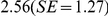 for Scenario 3.

### Power comparison between imputation-based meta-analysis and individual association studies

**Figure 3:**
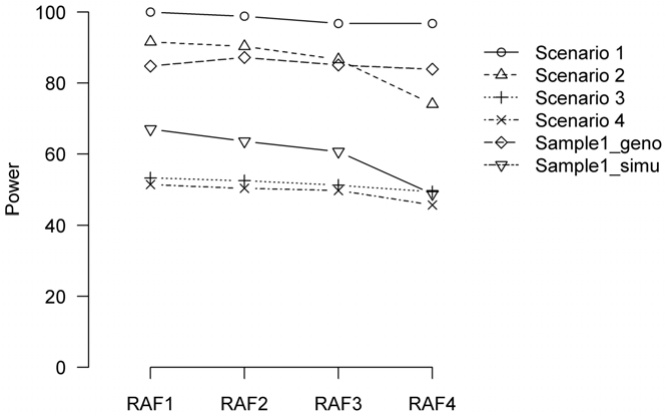
Power comparison between meta-analysis of different scenarios and association analysis in individual Sample 1. Sample1_geno and Sample1_impu refer to situations where causal SNPs are typed and imputed, respectively, in Sample 1. QTL variation of 2.0% is used.

Power comparisons between meta-analysis and individual association analyses were conducted and were partially shown in Fig. 3. As indicated previously, meta-analysis with causal SNPs typed (Scenario 1) or imputed (Scenario 2) across sub-samples had higher power. When all causal SNPs were typed in Sample 1, the power of the analyses in individual Sample 1 was compatible with that of Scenarios 1 and 2, and was higher than that of Scenarios 3 and 4. Moreover, the power of the analyses in individual Sample 1 was still mostly higher than that of Scenarios 3 and 4, even when causal SNPs were imputed in Sample 1. These results illustrate the importance of taking cautions when applying meta-analysis with genotype imputation to GWAS, as the power of the analyses in individual samples may not be necessarily lower than that of the meta-analysis with genotype imputation.

## Discussion

In this study, we investigated the performance of imputation-based meta-analysis of GWAS through an empirical GWAS genotype data. Considerable between-study heterogeneity was detected and reduction in detection power was observed when causal variants were typed in some individual studies and imputed in the others. Specifically, for meta-analysis using fixed-effects model, the power loss was up to ∼25% for situations that causal genes were genotyped in some but not all individual component studies, comparing to relatively homogenous situations of typed causal variants across all individual studies. Notably, for situations of partially directly-typed causal variants, the power of meta-analysis may be lower than that of analyses performed in some individual studies.

An important issue in meta-analysis is the potential existence of between-study heterogeneity, which can affect the power of the meta-analysis. In the context of genome-wide association studies, the between-study heterogeneity can be caused by various factors, such as the variation of genetic effects across different populations, incomparable measures of phenotype used in different studies, and/or the deviation from Hardy–Weinberg equilibrium for SNPs and population stratification. With the above-mentioned heterogeneity-causing factors removed by constructing our sub-samples from a single population and using the same distribution for genetic effects across sub-samples, the observed between-study heterogeneity in our simulated sub-samples will be most likely to be only due to imputation, indicating another possible source of between-study heterogeneity for the meta-analysis of GWAS.

Although our study is based on simulations for an empirical data set, the observations from our study are closely relevant to practical analyses. An interesting and useful observation is that a meta-analysis with imputation is not necessarily more powerful than that of analyses performed in individual samples, as shown in Fig. 3, especially when imputation was performed in some but not all individual samples. This may be because the loss of power due to the introduced uncertainty by imputation is greater than the gained power by increasing the sample size through meta-analysis. This observation illustrates the importance of taking cautions in the application and data interpretations of applying meta-analysis to GWAS. In addition, we compared the analyses under different imputation scenarios, one of which is that SNPs are genotyped in some individual samples, but untyped in other samples. This situation is not uncommon in practice. For example, when different genotyping platforms are used for different GWA studies, many SNPs will be genotyped in some samples for one specific genotyping platform, and be untyped in other samples with a different genotyping platform, requiring imputations in some but not all sub-samples. Another situation is when *in silico* replications are performed for candidate or genome-wide association analyses. In this case, statistically significant SNPs are genotyped in the discovery sample, and may not be genotyped in all the replication samples and thus need to be imputed, such as in our previous study [19].

Both risk increasing allele frequency and the magnitude of variation explained by the causal SNPs can affect between-study heterogeneity, as shown in Fig. 1. Briefly, for a specific value of *var*, an increasing RAF results in less between-study heterogeneity; and for a specific range of RAF, an increasing value of *var* results in more between-study heterogeneity. These trends may be partially explained by effect sizes of the causal SNPs. On one hand, greater effect sizes may imply greater between-population variation for the causal SNPs, and thus greater chances for observing between-study heterogeneity. On the other hand, it is known from population genetics theory that the variation explained by the causal SNPs is proportional to RAF*(1-*RAF*)*a ^2^, where *a* is the effect size for the causal SNP. Thus for a fixed value of *var*, an increasing RAF yields a smaller value for *a*; and for a specific range of RAF, an increasing *var* yields a greater value for *a*. For either case, the magnitudes of effect sizes are positively correlated with those of between-study heterogeneity.

To deal with the negative impacts of genotype imputation on meta-analysis of GWAS, we provide the following suggestions based on our study. Firstly, focus on SNPs presented in all individual studies, as meta-analysis with direct genotypes in all individual studies help to avoid generating spurious between-study heterogeneity by genotype imputation. Secondly, random-effects model should be used when significant between-study heterogeneity is detected. Although the random-effects model may not guarantee higher power than that of the fixed-effects model, it may help obtain more accurate effect size estimation. Thirdly, when sample sizes of individual studies vary largely, the results from the largest individual sample should be carefully evaluated, as it may provide better power than that of mate-analysis with imputation. Lastly, improving imputation accuracy may be useful in reducing between-study heterogeneity introduced by genotype imputation.

A number of issues in our studies may need further investigations. For example, only three GWAS sub-samples were used in our current study. Although a similar number of individual populations were used in various published meta-analysis of GWAS (e.g., [14], [20]), increasing the number of sub-samples in simulation studies may be needed in order to provide more robust conclusions. In our simulations, all sub-samples are constructed from the same genotyping platform. Additional simulations may be helpful in understanding the power for meta-analysis of GWAS using samples with different genotypes. Thus in future studies, we will perform analyses to investigate situations such as increased numbers of sub-samples and sub-samples with different genotyping platforms.

## Materials and Methods

In this section, we will first summarize how the sub-populations are constructed; we then describe the model and procedure for phenotype simulation; and at the end, we describe several topics related to our analyses, including imputation method, analytical models and test statistic for meta-analysis, and heterogeneity detection.

### Sub-population construction

The individual study samples used for the meta-analysis were constructed from an empirical GWAS dataset, the genome-wide genotyping data obtained from FHS SNP Health Association Resource (SHARe) project. The application for using the data has been approved by Tulane University Institutional Review Board and the access to the data has been granted by NHLBI Data Access Committee. The dataset contains more than 9,300 subjects from three generations of over 900 families and was genotyped for ∼550,000 SNPs (Affymetrix 500 K mapping arrays plus Affymetrix 50 K supplemental arrays). Detailed information about the FHS and its genotyping dataset can be found at the dbGaP website. For simplicity, we only used the SNPs in the 500 K array for subsequent analyses.

To imitate meta-analysis, three sub-samples (Samples 1–3) were constructed, with related information summarized in Table 1. Briefly, Sample 1 included all unrelated subjects from the 1^st^ generation (two at most in each pedigree), and married-ins in the 2^nd^ and 3^rd^ generations. Sample 2 and Sample 3 were constructed by randomly selecting one subject from the rest members of the 2^nd^ and 3^rd^ generations in each pedigree, respectively. This selection strategy helps to ensure unrelatedness among individuals within each sample. After data quality controls, including removing individuals with genome-wide genotype missing rates >10%, SNPs with genotyping call rates <90% or minor allele frequencies <0.01, and Hardy-Weinberg equilibrium test p-values< = 0.0001, the numbers of individuals for Samples 1–3 are, respectively, 2,023 (883 males and 1,140 females), 1,055 (471 males and 584 females), and 806 (362 males, 444 females), and the number of common SNPs across all three populations is 392,261. Notice that throughout the simulations, genotypes were fixed and obtained from the FHS data set directly, and phenotypes were simulated as described in the next session.

Genotypes of three independent populations were also generated through simulations using the software HAPGEN2. The sample sizes for the three populations were 2000, 1000, and 1000, respectively. The genotypes were generated based on the 1000 Genomes data provided by the software, and SNPs matching those in the Affymetrix 500 K mapping array were then selected to be the genotyped SNPs for the simulated populations. This simulation produced fully independent samples.

### Phenotype simulation

For a di-allelic quantitative trait locus (QTL), the risk allele and the alternative allele are denoted by 1 and 0, respectively. The frequencies for the two alleles are assumed to be *p* and *q* ( = 1−*p*), respectively. With an additive genetic effect of 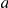, the phenotypic value for the *i*th individual is modeled by: 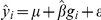, where 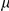 is the mean population phenotypic value, 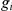 is the genotype score which is coded as the number of risk alleles carried by the *i*th individual, 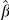 is the regression coefficient rendering the effect of the assessing QTL (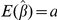), and 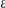 represents the residual error. The variance due to the QTL is then *var* or 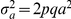.

To cover various biologically plausible conditions, our analyses were performed with a range of parameter values. Briefly, the variance explained by an assumed QTL was set as 0–2.0% with a 0.5% increment. Risk increasing allele frequency (RAF) was binned into four intervals: 0.01<RAF≤0.05, 0.05<RAF≤0.10, 0.10<RAF≤0.20 and 0.20<RAF≤0.50, which were represented by RAF1-4, respectively. To simulate meta-analysis with or without genotype imputation in individual studies, four scenarios (listed in Table 2) were considered: 1) causal SNPs were directly typed in all sub-samples; 2) causal SNPs were imputed in all sub-samples; 3) causal SNPs were imputed in Samples 1 & 2 and typed in Sample 3; and 4) causal SNPs were imputed in Sample 1 and genotyped in Samples 2 & 3.

The simulation process for phenotypic values follows the strategy proposed by Anderson et al [21]. Briefly, for each combination of parameter values, one SNP at each time was randomly picked as a causal variant from the genome-wide data set, and the phenotypic values were then simulated for the study subjects according to their genotypes for the SNP. The selected SNP was then set as directly typed or untyped. Power and type-I error were estimated as the proportions of significant simulation replicates with an assumed genome-wide significance level of 10^−7^ and an additive model. For each combination of RAF range and QTL variance, 1,000 simulations were performed.

### Genotype imputation and genetic association analysis in individual studies

Untyped SNPs were imputed by the program IMPUTE (Version 0.5.0) [16] using default parameters. Based on the hidden Markov Model and conditional on a set of known haplotypes and an estimated fine-scale recombination map, the program produces the probability distribution of missing genotypes. The phased HapMap II (rel#22 - NCBI Build 36) genotype data from the 60 CEU HapMap founders was used as the reference set. The minimum posterior probability of 0.95 was used as the threshold to accept the imputed genotypes as accurate for association tests [22].

SNP association tests were carried out by using the program SNPTEST (Version 1.5.1) [16], which implements an *F*-test and accounts for the uncertainty in the imputed genotypes.

### Meta-analysis

#### Fixed-effects and random-effects models

Fixed-effects model considers the genetic effects to be the same across all individual studies and assumes that any difference is due to chance. In genetic association studies, however, genetic effects could be different across populations due to various reasons such as allele frequency differences, different biases and estimation errors across studies. Thus to take these differences into consideration, random-effects model may be a better choice, as random-effects model assumes and can accommodate the potential differential effects across studies.

#### Test statistic for meta-analysis

In this study, we adopted the inverse variance method to construct the test statistic for meta-analysis, which was recently reviewed in the context of genetic association for quantitative traits by de Bakker and colleagues [5]. The test statistic takes the following form,
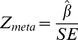
(1)where 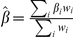, 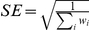, 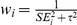. For fixed-effects model, 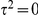, and for random-effects model, 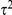 is a function of Cochran's Q (see below). 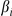 and 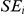 denote the beta coefficient and standard error of the estimated genetic effect in the *i*th study, respectively. The test statistic 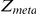 approximately follows a standard normal distribution, which is the basis for assessing its statistical significance.

#### Heterogeneity measurements

To test between-study heterogeneity, we used the *I* ^2^ index [6], which is
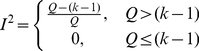
(2)where *k* is the number of studies and *Q* represents Cochran's *Q* statistic [23], defined as
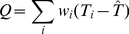
(3)where 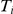 and 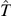 are effect sizes for the *i*th study and the combined study, respectively.

The 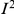 index, taking values between 0–100 percent, can be interpreted as a percentage of heterogeneity, that is, the part of total variation that is due to between-study variance. This statistic is independent of the number of studies and can be compared across meta-analyses with different number of studies and metrics.
